# Performance of Fully Automated Algorithm Detecting Bone Marrow Edema in Sacroiliac Joints

**DOI:** 10.3390/jcm12144852

**Published:** 2023-07-24

**Authors:** Joanna Ożga, Michał Wyka, Agata Raczko, Zbisław Tabor, Zuzanna Oleniacz, Michał Korman, Wadim Wojciechowski

**Affiliations:** 1 Department of Radiology, Jagiellonian University Medical College, ul. Botaniczna 3, 31-503 Krakow, Poland; michal.wyka@student.uj.edu.pl (M.W.); agata.raczko@student.uj.edu.pl (A.R.); zuzanna.oleniacz@student.uj.edu.pl (Z.O.); michal.korman@student.uj.edu.pl (M.K.); wadim.wojciechowski@uj.edu.pl (W.W.); 2 Department of Biocybernetics and Biomedical Engineering, AGH University of Science and Technology, al. Adama Mickiewicza 30, 30-059 Krakow, Poland; ztabor@agh.edu.pl

**Keywords:** deep learning, axial spondyloarthritis, sacroiliac joint, fully automated algorithm, coronal oblique plane

## Abstract

This study evaluates the performance of a fully automated algorithm to detect active inflammation in the form of bone marrow edema (BME) in iliac and sacral bones, depending on the quality of the coronal oblique plane in patients with axial spondyloarthritis (axSpA). The results were assessed based on the technical correctness of MRI examination of the sacroiliac joints (SIJs). A total of 173 patients with suspected axSpA were included in the study. In order to verify the correctness of the MRI, a deviation angle was measured on the slice acquired in the sagittal plane in the T2-weighted sequence. This angle was located between the line drawn between the posterior edges of S1 and S2 vertebrae and the line that marks the actual plane in which the slices were acquired in T1 and STIR sequences. All examinations were divided into quartiles according to the deviation angle measured in degrees as follows: 1st group [0; 2.2], 2nd group (2.2; 5.7], 3rd group (5.7; 10] and 4th group (10; 29.2]. Segmentations of the sacral and iliac bones were acquired manually and automatically using the fully automated algorithm on the T1 sequence. The Dice coefficient for automated bone segmentations with respect to reference manual segmentations was 0.9820 (95% CI [0.9804, 0.9835]). Examinations of BME lesions were assessed using the SPARCC scale (in 68 cases SPARCC > 0). Manual and automatic segmentations of the lesions were performed on STIR sequences and compared. The sensitivity of detection of BME ranged from 0.58 (group 1) to 0.83 (group 2) versus 0.76 (total), while the specificity was equal to 0.97 in each group. The study indicates that the performance of the algorithm is satisfactory regardless of the deviation angle.

## 1. Introduction

Axial spondyloarthritis (axSpA) is a group of rheumatic inflammatory conditions that affect the sacroiliac, intervertebral and facet joints [1]. The prevalence of axSpA ranges from nine to 30 per 10,000 people [2]. It is approximately two- to three-fold higher in men than in women [3]. In general, axSpA initially presents during the third decade of life [4]. The classification criteria for axial spondyloarthritis, developed by the Assessment in SpondyloArthritis international Society (ASAS), include the presence of sacroiliitis detected by radiography or by magnetic resonance imaging (MRI) with the addition of at least one SpA feature (“imaging arm”), or the presence of HLA-B27 with the addition of at least two SpA features (“clinical arm”) [5]. AxSpA results in persistent inflammation of the sacroiliac joints (SIJs), causing chronic back pain, stiffness, skeletal and postural changes. This condition limits the ability to perform daily living activities, which contributes to a negative effect that axSpA has on HRQoL (health-related quality of life) [5]. However, appropriate treatment, physical therapy, and psychosocial interventions can improve HRQoL [6].

Currently, magnetic resonance imaging (MRI) of sacroiliac joints is considered a first-line method of choice for early axSpA diagnostics [7], although other imaging modalities can also be used to diagnose abnormal conditions in the joints [8] This examination is performed only for specific indications, including bone, joint or soft tissue infection, inflammation of SIJ, SIJ dysfunction, ankylosing spondylitis, degenerative arthritis, SIJ pain or stiffness, avascular necrosis, rheumatoid arthritis, osteoarthritis, sacroiliitis, psoriasis and gout [9]. MRI is, therefore, a diagnostic procedure that enables the assessment of active inflammation within SIJs (sacroiliitis) [10,11].

This type of examination allows the evaluation of bone marrow edema (BME), erosions, fat lesions, sclerosis or ankylosis (bone formation) [12]. According to the ASAS classification criteria, active sacroiliitis in MRI is defined by the following:

1. Bone marrow edema (collection of fluid between bone marrow cells due to inflammation) visible on “fluid-sensitive” STIR sequence (Short Tau Inversion Recovery) (also known as T2-weighted sensitive to water sequence) as areas hyperintense to the sacral interforaminal bone marrow (brighter areas).
2. Bone marrow enhancement (osteitis), which is observed on T1-weighted sequence after contrast media administration [9,13,14].

To determine the presence of sacroiliitis in MRI, such changes must be clearly present, located in a typical anatomical area (subchondral bone), and apparent on at least two consecutive slices of examination, or more than one lesion must occur on the same slice [9,10,15]. The SPARCC (The Spondyloarthritis Research Consortium of Canada) scale is used for evaluating inflammatory lesions in the SIJs, measuring the presence, depth, and intensity of bone marrow inflammation [16]. 

Early diagnosis of axSpA is crucial to treatment results and the future quality of life of affected patients [17]. However, there is often perennial delay between the onset of symptoms and diagnosis [18,19].

Using the criteria of the European Spondyloarthropathy Study Group (ESSG) or Amor [20], patients who do not fulfill the criteria for AS but present with clinical features of SpA can be classified as undifferentiated SpA. Nevertheless, long-term studies suggest that more than 40% of patients in this group may not develop AS even after 10 years of follow-up. This clearly demonstrates the importance of early diagnosis, especially in the preradiographic phase when sacroiliac arthritis is not yet visible on radiographs [21], necessitating the use of other imaging techniques. Hence, the crucial role of using MRI in the early diagnosis of axSpA [22,23]. The main benefits of accelerating the diagnostic process are improved results of biological treatment [24] and prevention or delay of the onset progression in the form of structural damage [25].

Artificial intelligence (AI) can be defined as the ability of a computer system to perform tasks that would typically or traditionally require human intelligence [26]. Machine learning is a branch of artificial intelligence that allows the extraction of meaningful patterns from practical examples [27,28]. MRI analysis of patients with axSpA is a time-consuming process, requiring radiological specialized knowledge and evaluation experience. Streamlining this process can be possible through the use of artificial intelligence [29,30]. Up to now, only two algorithms for semi-automated detection and quantification of axSpA-related sacroiliitis have been developed—the first by Zarco et al. [31] and the other one by Kucybała et al. [12]. In the study of Rzecki et al. [30], a fully automated algorithm for BME evaluation was described. This fully automated algorithm was compared with other approaches in [32], further demonstrating its potential for clinical use. The following year, another algorithm for analyzing MR images of the SIJs in axial spondyloarthritis was developed by Bressem et al. [33]. In contrast to the algorithm described in [30], the algorithm of Bressem et al. [33] focuses only on classifying whole MR images as either normal or abnormal; that is, the location and the volume of the lesion are not determined using the algorithm [33]. 

The algorithm described in [30] was, however, developed and tested using a carefully selected dataset. During the selection, attention was paid to the high technical quality of the MR examination, which do not necessarily always meet everyday clinical conditions. Before the algorithm of [30] can be implemented in clinical settings, it must be demonstrated that it is not sensitive to typical variations in the MR acquisition protocol. For this reason, the aim of this study is to validate the performance of the fully automated algorithm [30] designed for BME evaluation depending on the technical correctness of the MRI examinations of the SIJs. 

## 2. Materials and Methods

### 2.1. Material

The study obtained approval from the Institutional Bioethics Committee (No. Of approval: 1072.6120.332. 2022, date of approval: 18 January 2023). This is a retrospective study of 173 patients who underwent MRI examinations of the SIJs. In this group, 47% (*n* = 82) of patients were male, while 53% (*n* = 91) of patients were female. The median age of the patients was 31, with an IQR of 23 years (range 18–86 years). All of them underwent sacroiliac joint MRI due to suspected axSpA. Therefore, the patients population is very diverse and among them there are people without confirmed axSpA, people in the early stages of the axSpA, and those who were found to have irreversible changes after examination. Random selection of patients was crucial in order to evaluate the performance of the algorithm, as it best reflects the population that the algorithm will deal with if implemented as a tool used in daily practice by radiologists or other specialists. The same applies to the verification of the algorithm with regard to the technical correctness of the MRI examinations of the SIJs.

All analyses were based on processing T1-weighted, T2-weighted, and STIR sequence images. T2-weighted sequences were acquired in the sagittal plane, while T1-weighted and STIR sequences were in the coronal oblique plane, and the position of the patient remained unchanged during their acquisition time. The examinations involved in the present research were performed using a 3.0 Tesla MRI scanner (Achieva, Philips Healthcare, Amsterdam, The Netherlands) and an 8-channel phased-array XL-torso body matrix coil.

Detailed scan parameters were as follows:

- For T1-weighted turbo spin echo (TSE) sequence—TR 500 ms, TE 14 ms, flip angle 90, NEX 1, slice thickness 3 mm, matrix 560 × 560, FOV 240 × 240 × 71,
- For STIR TSE sequence—TR 5239 ms, TE 30 ms, inversion time 190 ms, flip angle 90, NEX 2, slice thickness 3 mm, matrix 400 × 400, FOV 240 × 240 × 71.

In each MRI examination, the number of slices in each series varies between 12 and 24.

### 2.2. Methods

In order to properly evaluate the performance of the algorithm depending on the technical correctness of the MRI examination of the sacroiliac joints, this study consisted of the following steps:

1. Assessment of the correctness of the alignment of the MRI sections of the SIJs.
2. Enhancement of the pre-existing algorithm in the form of post-processing adjustments.
3. Manual and automatic segmentation of the sacrum and iliac bones.
4. Evaluation of BME using the SPARCC scale.
5. Manual and automatic segmentation of BME.
6. Statistical analysis of the results.

#### 2.2.1. Assessment of the Correctness of the Alignment of the MRI Section of the SIJs

In order to standardize the assessment, the researchers decided to create an angle, which will determine the technical correctness of the entire examination. This angle is called the deviation angle and is located between the fully correct line drawn between the posterior edges of the S1 and S2 vertebrae and the line that marks the actual plane in which the slices were acquired in the T1 and STIR sequences. Owing to this method, the researchers could verify how the fully automated algorithm performs in real-life conditions. The greater the deviation angle measured by the investigators, the lower the quality of the slices in T1-weighted and STIR sequences. Slices obtained in an inaccurate plane do not fully visualize the sacroiliac joints, which impairs their assessment.

**Figure 1:**
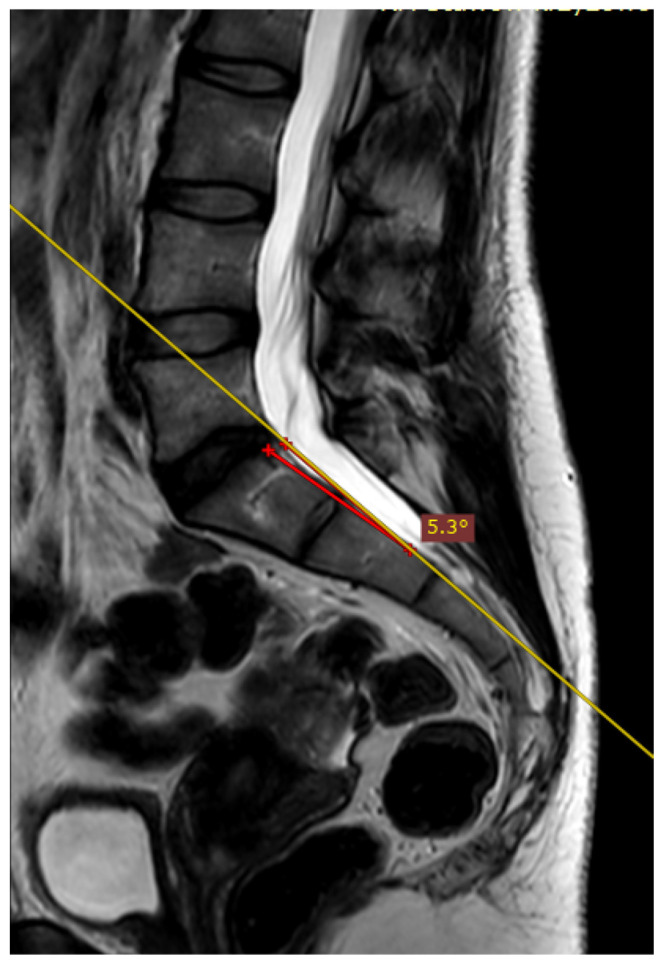
The measured deviation angle equals 5.3 degrees (group 2). The red line is the fully correct line drawn between the posterior edges of the S1 and S2 vertebrae. The yellow line marks the actual plane in which the slices were acquired.

**Figure 2:**
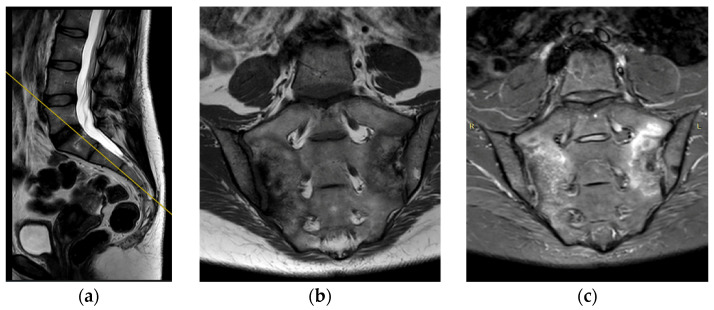
(**a**) Example of sagittal T2 series. Yellow line indicates the position of coronal projections (**b**,**c**). (**b**) Example of coronal T2 series. (**c**) Example of coronal STIR series.

The deviation angles were measured on T2-weighted sequence slices obtained in the sagittal plane, as shown in Figure 1. Figure 2, presented below, shows the interrelationship of the three sequences of the same examination (as shown in Figure 1) that were used in this study. The RadiAnt DICOM Viewer was used to take measurements. All examinations were analyzed by two independent researchers in a blind study. The final results of the measurements were averaged to obtain a single deviation angle corresponding to each examination.

Furthermore, all examinations have been divided into four groups based on the quartile values of the angles measured in degrees, ensuring that the groups have similar sizes. The criteria for dividing the examinations into groups are listed below:

1. Angle dimensions [0; 2.2].
2. Angle dimensions (2.2; 5.7].
3. Angle dimensions (5.7; 10].
4. Angle dimensions (10; 29.2].

#### 2.2.2. Enhancement of the Pre-Existing Algorithm in the Form of Post-Processing Adjustments

To perform the detection of the first sacrum and iliac bones, and then the BME, the fully automated algorithm described previously was used [12,30]. The algorithm, described in detail in the study of Kucybała et al. [12] and Rzecki et al. [30], creates segmentations of three regions corresponding to both the iliac bones and sacrum in T1-weighted sequences, and then these segmentations are superimposed on STIR sequence so that the algorithm can perform segmentations of the BME. The transfer of bone segmentations from T1-weighted to STIR-weighted sequences is feasible due to the assumption that the patient’s position was unchanged during the acquisition of both sequences.

Post-processing adjustments were implemented to adapt the performance of the algorithm [30] to the guidelines [9,10,15], which state that an inflammatory lesion can be identified if it is present on two consecutive MRI slices or if there is more than one lesion on a single slice. In addition, the inflammatory lesion must be located in typical anatomical areas, such as subchondral or periarticular bone marrow [9]. 

#### 2.2.3. Manual and Automatic Segmentation of the Sacrum and Iliac Bones

**Figure 3:**
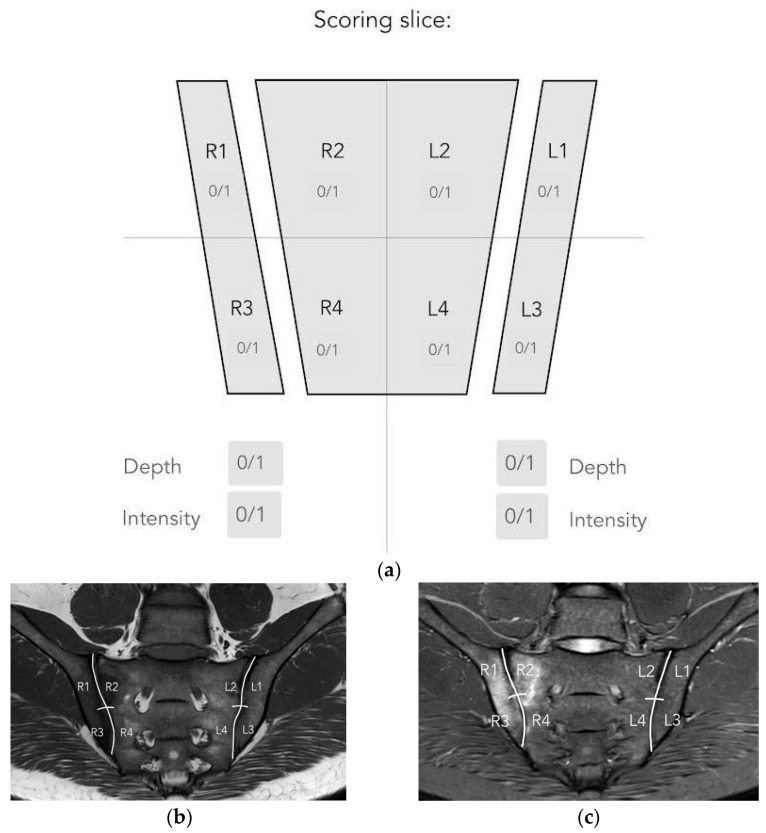
Scoring scale used in the study. The evaluation involves six slices of each examination. (**a**) Graphic illustrating the division of the SIJ into quadrants: R1—right iliac (upper), R2—right sacral (upper), R3—right iliac (lower), R4—right sacral (lower), L1—left iliac (upper), L2—left sacral (upper), L3—left iliac (lower), L4—left sacral (lower). Depth and intensity rating details (SPARCC scale) are provided below the graphic. (**b**) Quadrant division illustrated on the T1 sequence. (**c**) Corresponding quadrant division illustrated on the STIR sequence.

In order to compare the performance of the improved algorithm depending on the technical correctness of the MRI of the sacroiliac joints, it is necessary to perform both manual and automatic bone segmentations. Therefore, it was decided to manually segment the iliac and sacral bones and then evaluate their agreement with the segmentations created by the algorithm. The area of the right and left iliac bones, as well as the sacrum, were manually labeled on consecutive slices of the T1-weighted sequence by one researcher. The segmentations were prepared manually with the 3D Slicer (version 4.11.20210226) program and then verified by two other members of the research team. After the verification, it was stated that the created segmentations cannot be more accurate and can serve as a reference for segmentations performed by the algorithm. The establishment of reference segmentation considerably simplifies the process of comparing algorithm performance, as it provides more tools that can assess the compatibility of human and artificial intelligence. The compatibility of both segmentations was evaluated by several means. Initially, to have all bone segmentations manually reviewed one by one by the researchers, a proprietary scale was introduced to assess compliance. The scale is shown in Figure 3 and was based on the SPARCC scale. In this visual scale, each examination can receive a maximum score of 48 points. Only six slices that represent the largest surface of the SIJs are selected and afterwards evaluated. Each SIJ is divided into quadrants. In each quadrant, the correspondence of manual and fully automated segmentation is evaluated; if both segmentations correspond, one point is granted. Secondly, the Dice coefficient (DC) is calculated as follows:

$$\mathrm{DC}=\frac{2\left|M\cap^{\;}A\right|}{\left|M\right|+\left|A\right|}$$

where M and A stand for manually and automatically segmented bone regions, respectively, while |X| for some region X denotes the volume (number of voxels) of X.

#### 2.2.4. Evaluation of BME Using the SPARCC Scale

To assess the presence, depth, and intensity of BME, the SPARCC scale was used. Only six selected slices representing the largest proportion of the SIJ are evaluated. Points are assigned dichotomously: present—1, absent—0. Each SIJ is divided into four quadrants: 1 upper iliac, 2 lower iliac, 3 upper sacral, 4 lower sacral (Figure 3). The presence of increased signal in each quadrant is recorded. The maximum score for two joints in each slice is eight. The maximum score for the whole examination is 48. All lesions presented in the iliac bone are evaluated, whereas in the sacrum, lesions medially extend as far as the lateral border of the sacral foramina. A score for “intensity” can be granted separately for each of the SIJs on each slice. The reference for awarding a point for intensity is high signal from slow-flowing venous blood within presacral veins. One point is awarded when the “intense” signal is present in at least one quadrant of the joint on a single slice. The maximum score for one slice is two, therefore for six slices it is 12. A score for “depth” can be granted separately for each of the SIJs on each slice. A lesion is classified as “deep” if there is a homogeneous and unambiguous increase in signal extending over a depth of at least 1 cm from the articular surface. One point is awarded when the “deep” signal is present in at least one quadrant of the joint on a single slice. The maximum score for one slice is two, therefore for six slices it is 12 [14].

The total maximum score is 72:

1. Presence of bone marrow edema = 48.
2. Presence of intense edema = 12.
3. Presence of deep edema = 12.

All examinations were analyzed by two researchers who did not contact each other during the SPARCC scale completion. After obtaining the scores, the results of the two researchers were compared, and examinations that were rated unequally were submitted to a consortium composed of all of members of the research team, who collectively decided on the ultimate scores. 

#### 2.2.5. Manual and Automatic Segmentation of BME

The manual segmentations of BME based on SPARCC scale scores were completed for each examination using the 3D Slicer (version 4.11.20210226) program. Segmentations were obtained on all slices of the STIR sequence of each examination. Once all the manual segmentations were produced, each segmentation was further verified by three researchers for complete accuracy and consistency. This method of creating reference segmentations was adopted because the evaluation of lesions is subjective (meaning that the ground truth is burdened with uncertainty, making the problem difficult). Therefore, it was decided that the review performed by a total of three researchers can indeed be considered reliable. The resulting segmentations were consensually recognized as reference segmentations to be subsequently used for verification of automatic segmentations. The evaluation of segmentations performed manually and those performed by the automated algorithm has also been verified in several ways. The first step was a semi-quantitative assessment based on a scale created by the researchers (Figure 3), similar to the scale used previously to evaluate the correspondence of the manual and automated segmentation of sacral and iliac bones. The maximum score was 48 points, with one point granted for each quadrant of both joints if the manual reference and automated segmentations equally detected or excluded inflammatory lesion. Due to this scale, all segmentations of inflammatory lesions were required to be viewed by the researchers. 

In addition to this visual scale, both versions of segmentations were further compared in a quantitative manner. The automated segmentation of lesions assigned a real number to every voxel within the volume of interest, which can be interpreted as a probability that the voxel is within a lesion region. Then, if the probability is larger than some user-selected decision threshold, the voxel receives a lesion labeled. Otherwise, it receives a label of healthy tissue. Based on this labeling, the evaluation is further based on counting the voxels of true positive (TP), true negative (TN), false positive (FP) and false negative (FN). Voxels corresponding to areas of inflammatory lesions marked by both manual and automatic segmentations were considered true positive. Truly negative areas were those where the inflammatory lesions were not covered by any segmentation. On the basis of the above data, specificity, accuracy and sensitivity were calculated.

The selection of the decision threshold mentioned above is usually based on the analysis of the ROC (Receiver Operating Characteristic). To generate such curves, the decision threshold is varied from some low to some high value. Then, TP, TN, FP and FN are counted, and from these numbers, the false positive rate (FPR) and true positive rate (TPR) are calculated. The ROC curve is the plot of TPR vs. FPR. The area under the ROC curve (AUC ROC) is a measure of automated classification accuracy, with AUC ROC equal to one for a hypothetical ideal classification algorithm. The optimal decision threshold can be derived from the ROC curve, for example, as the one that maximizes the sum of specificity and sensitivity [12].

#### 2.2.6. Statistical Analysis of the Results

Since the studies were divided into groups on the basis of the mean measured deviation angle of the two raters, the intraclass correlation coefficient (ICC) was calculated to test the absolute agreement of those raters. The ICC (2, 2) estimate and its 95% confidence interval were calculated based on the mean rating (k = 2), absolute agreement, and the two-way random effects model.

Statistical analysis was then applied to the bone and BME segmentations, visual scale results, and SPARCC scores. In particular, they were checked for differences in groups based on the deviation angle value using the Kruskal-Wallis H test due to the rank character of those scales. Additionally, the Dice coefficient (DC) was calculated on the basis of comparison between algorithm prediction and the manually created mask (ground truth) and analyzed in terms of group differences also using the Kruskal-Wallis H test. However, this coefficient was calculated only for bone segmentations due to its limitations when describing small structures [34]. Algorithm predictions for BME were evaluated with the use of ROC. ROC curves were calculated for all the samples and separately for the samples assigned to the four groups differing by the technical correctness of the MRI examination. Additionally, specificity, sensitivity and accuracy based on total number of voxels in algorithm predictions indicated as TP, TN, FP or FN when comparing with manual segmentations of BME were calculated, assuming the decision threshold equal to 0.5. Sensitivity based on the count of single lesions detected by the algorithm was also calculated.

Statistical analysis was performed using the Stata/SE 17.0 package with the assumption of a significance level at 0.05.

## 3. Results

### 3.1. Assessment of the Correctness of the Alignment of the MRI Section of the SIJs

**Figure 4:**
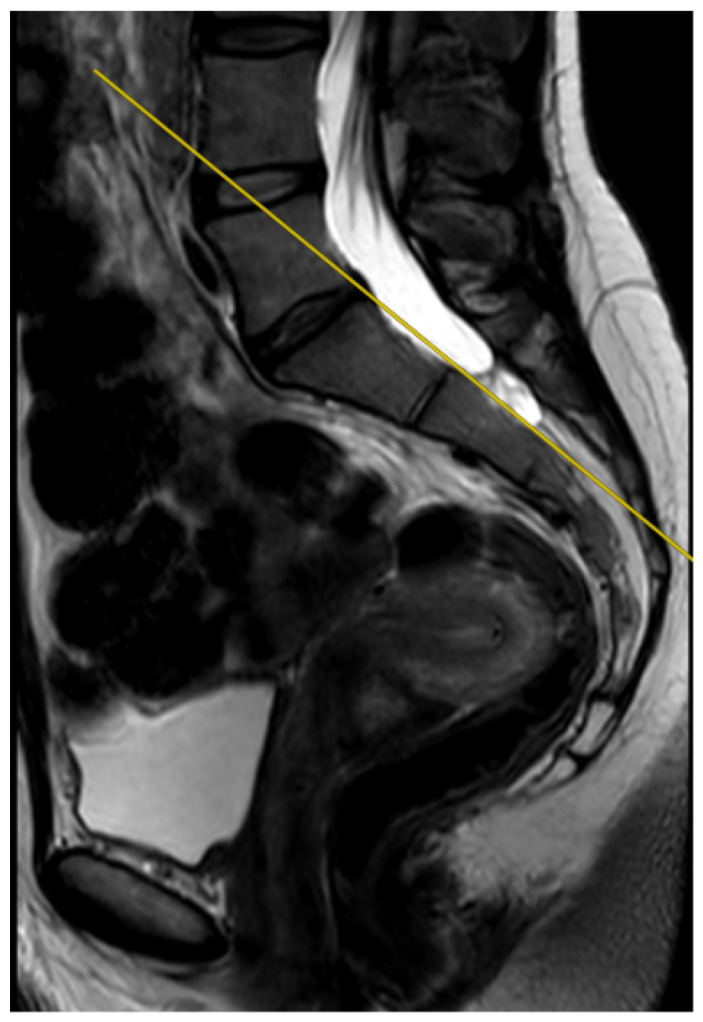
The measured deviation angle equals 0 degrees, properly performed MRI examination of the sacroiliac joints, T2-weighted sequence. The yellow line marks the actual plane in which the slices were acquired, while it is also the fully correct line drawn between the posterior edges of the S1 and S2 vertebrae.

**Figure 5:**
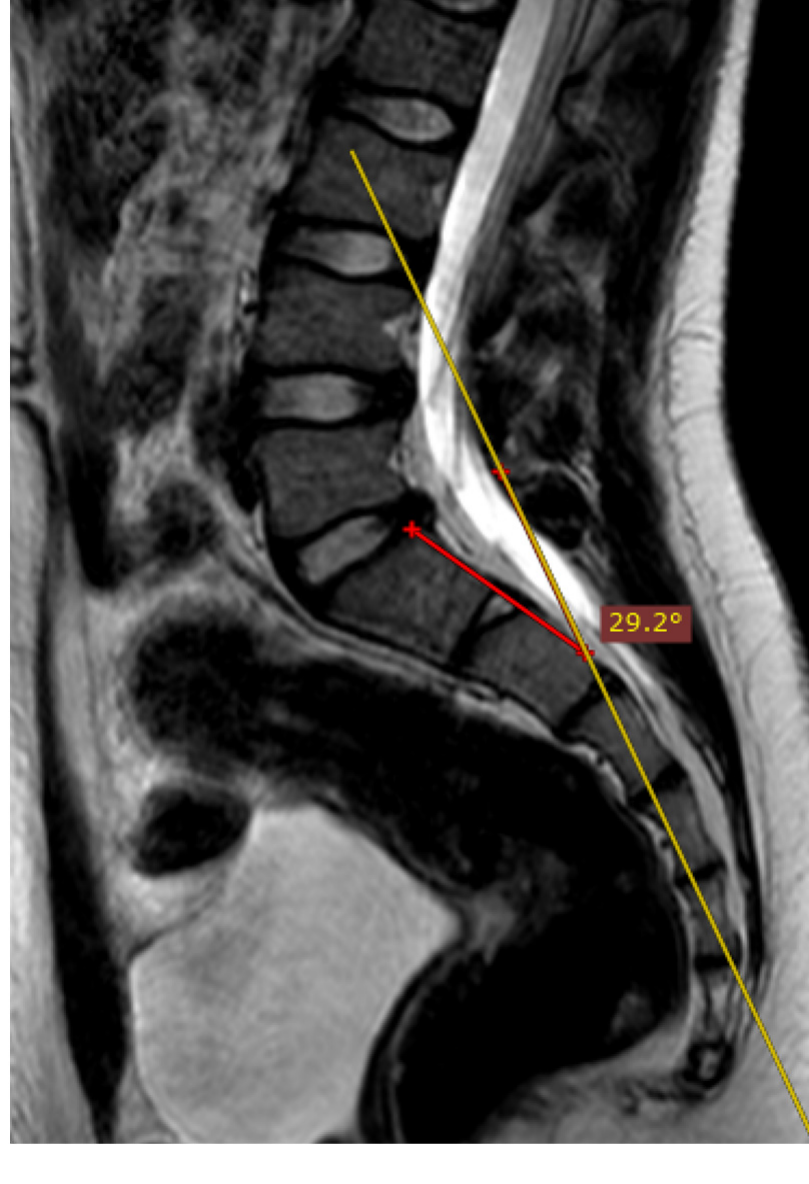
The measured deviation angle equals 29.2 degrees (group 4), T2-weighted sequence. The red line is the fully correct line drawn between the posterior edges of the S1 and S2 vertebrae. The yellow line marks the actual plane in which the slices were acquired.

**Figure 6:**
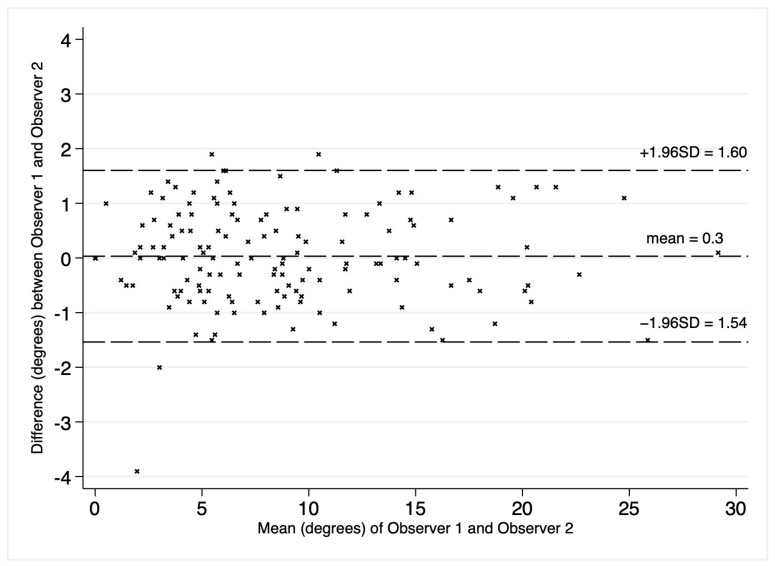
Difference between measurements of deviation angle (degrees) in 173 studies taken by Observer 1 and Observer 2 plotted against their mean.

Overall, 20% (*n* = 35) of the examinations included in the study were performed perfectly correctly considering the technical aspect of the MRI procedure. The examination was defined as fully correct if the deviation angle measured by all of the researchers equaled 0 degrees (Figure 4). The median deviation angle was equal to 5.7 degrees. The maximal deviation angle measured was 29.2 degrees (Figure 5). The ICC (2, 2) was computed as 0.9960 (95% CI [0.9945, 0.9970]), which indicates excellent reliability between the researchers when measuring the deviation angle. Additionally, Figure 6 represents a Bland-Altman diagram with the limits of 95% agreement stated. The results of the division into quartiles due to the deviation angle measurements of all the examinations are shown in Table 1. The obtained division was considered to be valid for the whole study, and on its basis the analysis of the subsequent parts of the study was performed.

### 3.2. Manual and Automatic Segmentation of the Sacrum and Iliac Bones

**Figure 7:**
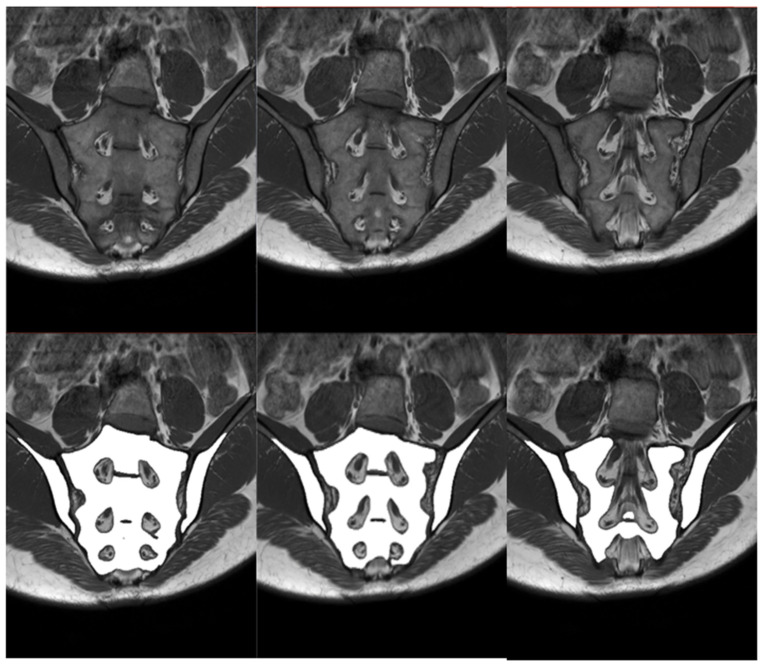
Sample slices of an average case (visual scale equal to 47, DC = 0.98): T1 sequence in the upper row, manual segmentation in the middle row and automated segmentation in the lower row.

The results of the segmentation of the sacrum and iliac bones are shown in the Table 2. The score awarded by the researcher was calculated for each examination and ranged from 0 to 48 points, with 48 points were awarded in the absence of uncertainty about the complete validity of the automatic segmentation. Fourteen percent (*n* = 25) of examinations were given 48 points, while the minimum score amounted to 26 points. The median of scores was equal to 47 (IQR, 5). Samples of the manual and automatic segmentations of the sacrum and iliac bones are presented in Figure 7. Kruskal–Wallis H test (H(3) = 0.512, *p* = 0.9162) was not statistically significant when comparing the results of the visual scale between groups, which suggests no differences in algorithm performance depending on deviation angle value.

The Dice similarity coefficient for comparison between manual and automatic segmentations, averaged over 173 examinations, ranged from 0.9277 to the maximum of 0.9968 (Table 2). The mean value of DC was 0.9820 (95% CI [0.9804, 0.9835]). As shown in Table 2, the mean value of Dice similarity coefficients varied between the groups from 0.9795 (95% CI [0.9758, 0.9832]) in the 3rd group to 0.9839 (95% CI [0.9807, 0.9871]) in the 1st group. Analysis in terms of group differences of DC with Kruskal–Wallis H test yielded a statistically significant difference between groups, H(3) = 8.362, *p* = 0.0391. Post-hoc comparison with the use of Dunn’s test with Bonferroni adjustment revealed a difference between group one and group three, which was considered statistically significant (*p* = 0.0166).

### 3.3. Evaluation of BME Using the SPARCC Scale

The final results of the SPARCC scale assessment are shown in Table 3. It is worth noting that statistical analysis showed no differences between groups in SPARCC scale points, which indicates homogeneity of groups in terms of the advancement of BME. The difference of SPARCC points was also checked using the Kruskal–Wallis H test, and the result was not statistically significant, H(3) = 0.754, *p* = 0.8604.

### 3.4. Manual and Automatic Segmentation of BME

**Figure 8:**
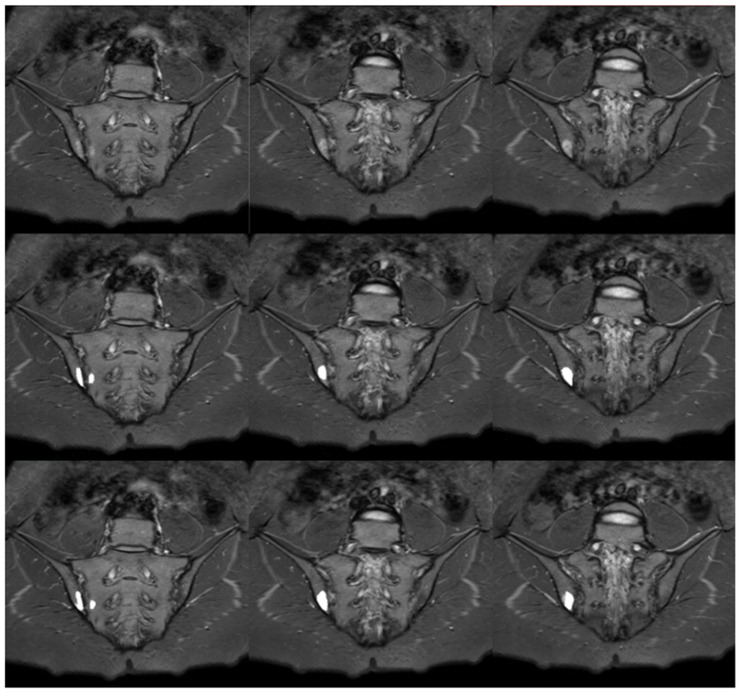
Sample slices with inflammatory lesions (SPARCC = 17): STIR sequence in the upper row, manual segmentation in the middle row and automated segmentation in the lower row.

The sample comparison of the performance of manual and automatic segmentations of BME is presented in Figure 8. The results of assessing the compatibility of manual and automatic BME segmentations with the visual scale described previously are provided in Table 4. Differences between the groups were checked using Kruskal–Wallis H test, which resulted in not being statistically significant, H(3) = 1.125, *p* = 0.7710. This outcome implies no difference in the algorithm’s performance in BME segmentation in relationship to the deviation angle. Due to the limitations of the SPARCC scale, the number of examinations which points were awarded (SPARCC > 0, *n* = 68) differs from the number of examinations with BME recognized when preparing manual segmentations (*n* = 83).

**Figure 9:**
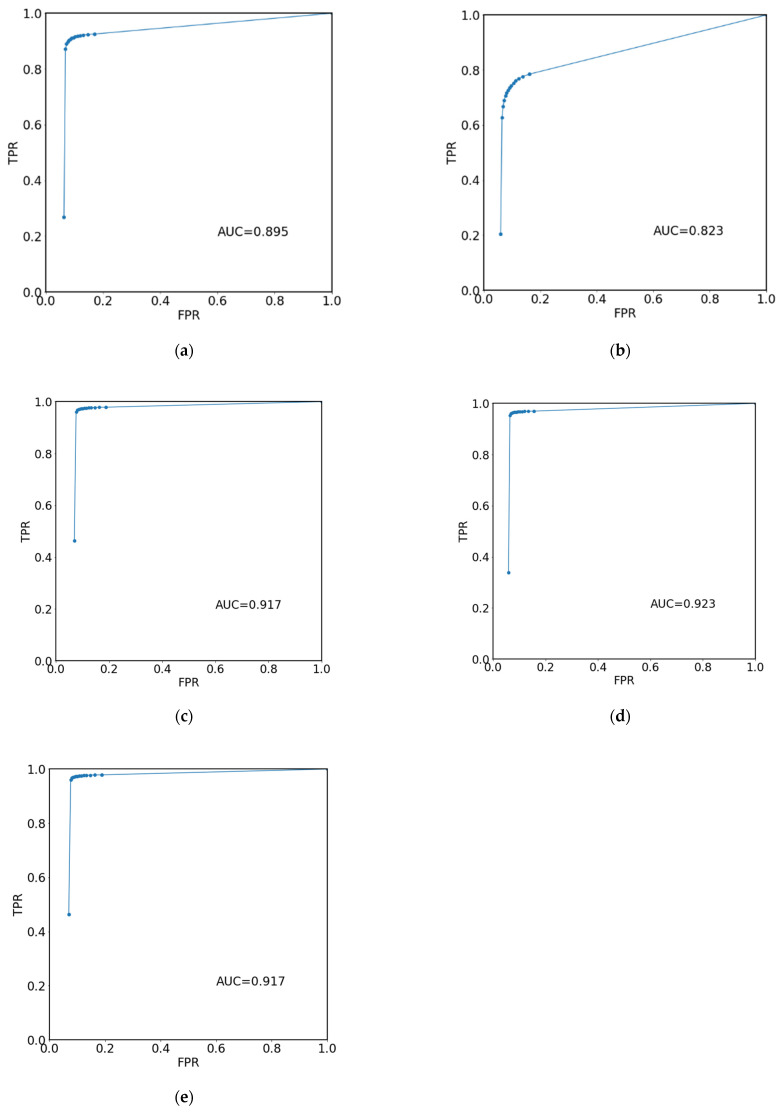
ROC curves calculated for all the samples (**a**) and separately for the samples assigned to the four groups: (**b**) Group 1; (**c**) Group 2; (**d**) Group 3; (**e**) Group 4.

Table 5 summarizes the results of the visual scoring scale with the division of each joint into quadrants. These results suggest that the algorithm underperforms when searching for lesions in quadrants lying on the sacral bone, due to higher values of interquartile range. Computed Receiver Operating Characteristics (ROC) curves are presented in Figure 9. The area under the curve (AUC) for all examinations is equal to 0.895. In the selected groups, the highest values were in groups 2 (AUC = 0.917) and 3 (AUC = 0.923), while the smallest in group 1 (AUC = 0.823) and group 4 (AUC = 0.887).

In order to further evaluate the performance of the algorithm, sensitivity, specificity and accuracy were also calculated basing on the total sum of voxels recognized as TP, TN, FP and FN. Moreover, the sensitivity for recognizing singular lesions was calculated. The results of these calculations are included in Table 6.

### 3.5. Summary of the Results

The most important data that summarize the performance of the algorithm is presented in Table 7.

## 4. Discussion

The technical aspect of performing sacroiliac joint MRI is often omitted in research studies. For radiologists to correctly evaluate the examination, it is crucial that the obtained images adequately represent the joints. Due to the anatomy of the pelvic and iliac bones, improper adjustment of the scanning plane can result in obtaining a completely different image than expected. For this reason, when developing algorithms aimed at assisting clinicians in the evaluation of SIJs, it is crucial to also retest them on examinations that were not performed completely appropriately in the technical aspect. The angle of deviation was selected as a method of assessing the technical correctness of the examination, since it determines the visual appearance of the obtained slices in the STIR sequence, which is used to assess BME.

Dividing all examinations into equal groups on the basis of the deviation angle enabled verification of the fully automated algorithm’s performance under various parameters. In addition, each group was similar in terms of the number of studies in which SPARCC was 0 or was >0 (Table 3). The results of the work demonstrated that despite the possibility that the algorithm could perform less properly with examinations that are not fully correct, it performs equally satisfactorily regardless of the deviation angle.

None of the previously developed semi-automatic or fully automatic algorithms [12,30,31,33] created for BME detection in the SIJs have not been tested on such a large number of examinations and against a technical correctness of the examination. Precisely for this reason, it makes the improved algorithm presented in this paper superior to previous ones. It is worth noting that machine learning algorithms’ performance critically depends on the size of the sample used during training, on the basis of which artificial intelligence “learns” to recognize a particular element. Therefore, it can create a sort of pattern that may eventually serve to distinguish pathology from normality [35]. Hence, the more extensive collection of examinations is assembled, the better the results achieved by the algorithm.

In order to segment BME in general, it is obligatory to correctly perform the bone segmentation. Therefore, it is essential for the fully automated algorithm to first achieve quality bone segmentation results, such as those reached in this study, as the average DC score is 0.9820 (95% CI [0.9804, 0.9835]), and even the minimum DC = 0.9277, which is also satisfactory. Statistical analysis does not disclose significant differences in DC values between groups one and three; however, obtained values of DC in all groups are high enough to assume that the algorithm detects bones accurately irrespective of the deviation angle.

Segmentation of BME is performed by the algorithm only on the region of bones marked in the previous segmentation. For this reason, if bone segmentation is performed correctly regardless the technical correctness of the examination, segmentation of BME as well will be performed within the relevant regions.

In order to compare manual and automatic segmentation of inflammatory lesions, not only a visual scale based on the SPARCC scale was used but also a mathematical evaluation of voxels, which eliminated the limitation of the visual scale resulting from the selection of only six slices in each examination. However, it is important to remember that the visual evaluation of the segmentations is essential since as of now, the radiologists’ expertise is considered to be the ground truth. 

Compared to the algorithm created by Bressem [33], the algorithm developed by our research team allows not only to globally determine whether a lesion is present in a given study but also to locate it in a specific quadrant of SIJs. In addition, what distinguishes our algorithm from the other one [33] is the ability to measure the size of the BME. This may prove useful in further studies on the correlation of inflammatory lesions with symptom severity in patients with axial spondyloarthritis. 

With this study, the fully automated algorithm for detecting BME was successfully validated on a number of examinations at least five times greater than the number of examinations on which it was initially created. It is worth mentioning that the age of the patients included in the study ranged from 18 to 86 years, which allows to conclude that the algorithm may have clinical application in the detection of BME in adults.

In conclusion, the fully automated algorithm remains competent to satisfactorily evaluate inflammatory changes in MRI examinations of the sacroiliac joints regardless of the deviation angle differences described. In addition, the algorithm was demonstrated to be useful for the exclusion of BME. 

## Figures and Tables

**Table 1:** The results of the division of all examinations into quartiles.

		Age			Deviation Angle (Degrees)		
		Median (IQR)	Min	Max	Median (IQR)	Min	Max
Total	Females (*n* = 91)	33 (18)	10	86	5.9 (9.8)	0	25.9
	Males (*n* = 82)	29 (22)	8	64	5.65 (6.6)	0	29.2
	*n* = 173	31 (23)	8	86	5.7 (7.8)	0	29.2
Group 1	Females (*n* = 25)	36 (18)	10	86	0 (0)	0	2.1
	Males (*n* = 19)	36 (28)	8	63	0 (0)	0	2.2
	*n* = 44	36 (20)	8	86	0 (0)	0	2.2
Group 2	Females (*n* = 19)	41 (22)	15	67	4.1 (1)	2.7	5.7
	Males (*n* = 24)	28 (16.5)	10	57	4.9 (1.8)	2.6	5.7
	*n* = 43	30 (25)	10	67	4.4 (1.8)	2.6	5.7
Group 3	Females (*n* = 20)	29.5 (19.5)	12	55	7.85 (2.7)	5.8	9.9
	Males (*n* = 23)	29 (22)	11	64	8.4 (2.4)	6.0	10.0
	*n* = 43	29 (21)	11	64	8.4 (2.6)	5.8	10.0
Group 4	Females (*n* = 27)	30 (21)	13	68	14.9 (5.5)	10.5	25.9
	Males (*n* = 16)	27 (26)	11	52	14.45 (8.35)	11.2	29.2
	*n* = 43	28 (22)	11	68	14.8 (6.9)	10.5	29.2

**Table 2:** The results of the comparison between manual and automatic segmentations of the sacrum and iliac bones with the use of the visual scale (score 0–48) and Dice similarity coefficient.

Groups Based on Deviation Angle	Visual Scale			Dice Similarity Coefficient			
	Median (IQR)	Min	Max	Mean (SEM)	95% CI	Min	Max
Total	47 (5)	26	48	0.9820 (0.0008)	[0.9804, 0.9835]	0.9277	0.9968
Group 1 [0; 2.2]	47 (4)	34	48	0.9839 (0.0016)	[0.9807, 0.9871]	0.9340	0.9968
Group 2 (2.2; 5.7]	47 (4)	26	48	0.9825 (0.0012)	[0.9801, 0.9848)	0.9564	0.9962
Group 3 (5.7; 10]	46 (5)	32	48	0.9795 (0.0019)	[0.9758, 0.9832]	0.9277	0.9958
Group 4 (10; 29.2]	47 (5)	32	48	0.9819 (0.0014)	[0.9793, 0.9846]	0.9584	0.9960

**Table 3:** The results of SPARCC scale assessment.

Groups Based on Deviation Angle		SPARCC Scale								
		Median (IQR)	Minimal Value	Maximal Value	SPARCC = 0	SPARCC > 0	SPARCC DEPTH = 0	SPARCC DEPTH > 0	SPARCC INTENSITIVITY = 0	SPARCC INTENSITIVITY > 0
Total	Females (*n* = 91)	0 (5)	0	49	54	37	80	11	61	30
	Males (*n* = 82)	0 (6)	0	56	51	31	70	12	52	30
	*n* = 173	0 (5)	0	56	105	68	150	23	113	60
Group 1 [0; 2.2]	Females (*n* = 25)	0 (3)	0	48	14	11	21	4	15	10
	Males (*n* = 19)	0 (4)	0	20	10	9	15	4	11	8
	*n* = 44	0 (6.5)	0	48	24	20	36	8	26	18
Group 2 (2.2; 5.7]	Females (*n* = 19)	1 (6)	0	45	9	10	17	2	12	7
	Males (*n* = 24)	0 (6.5)	0	51	17	7	19	5	17	7
	*n* = 43	0 (6)	0	51	26	17	36	7	29	14
Group 3 (5.7; 10]	Females (*n* = 20)	0 (4)	0	49	13	7	16	4	14	6
	Males (*n* = 23)	0 (10)	0	56	15	8	20	3	15	8
	*n* = 43	0 (9)	0	56	28	15	36	7	29	14
Group 4 (10; 29.2]	Females (*n* = 27)	0 (5)	0	25	18	9	26	1	20	7
	Males (*n* = 16)	0 (4)	0	31	9	7	16	0	9	7
	*n* = 43	0 (4)	0	31	27	16	42	1	29	14

**Table 4:** The results of the assessment of the compatibility between manual and automatic BME segmentations.

Visual Scale			
Groups Based on Deviation Angle	Median (IQR)	Min	Max
Total	43 (7)	22	48
Group 1 [0; 2.2]	42.5 (9)	22	48
Group 2 (2.2; 5.7]	43 (8)	23	48
Group 3 (5.7; 10]	43 (8)	25	48
Group 4 (10; 29.2]	45 (7)	18	48

**Table 5:** The summary of the results of visual scale assessment with the division into quadrants of each SIJ.

Groups Based on Deviation Angle	Median for the Sum of Points Scored on Six Slices in Each Quadrant			
	(IQR—Interquartile Range)			
Total	Right Joint		Left Joint	
	Upper iliac quadrant	Upper sacrum quadrant	Upper sacrum quadrant	Upper iliac quadrant
	6 (0)	6 (1)	5 (2)	6 (0)
	6 (0)	6 (1)	5 (3)	6 (1)
	Lower iliac quadrant	Lower sacrum quadrant	Lower sacrum quadrant	Lower iliac quadrant
Group 1 [0; 2.2]	Right joint		Left joint	
	Upper iliac quadrant	Upper sacrum quadrant	Upper sacrum quadrant	Upper iliac quadrant
	6 (0)	6 (1)	4.5 (2)	6 (0)
	6 (0)	6 (1.5)	5 (3)	6 (1)
	Lower iliac quadrant	Lower sacrum quadrant	Lower sacrum quadrant	Lower iliac quadrant
Group 2 (2.2; 5.7]	Right joint		Left joint	
	Upper iliac quadrant	Upper sacrum quadrant	Upper sacrum quadrant	Upper iliac quadrant
	6 (0)	6 (2)	5 (2)	6 (0)
	6 (1)	6 (2)	5 (3)	6 (2)
	Lower iliac quadrant	Lower sacrum quadrant	Lower sacrum quadrant	Lower iliac quadrant
Group 3 (5.7; 10]	Right joint		Left joint	
	Upper iliac quadrant	Upper sacrum quadrant	Upper sacrum quadrant	Upper iliac quadrant
	6 (0)	6 (1)	5 (2)	6 (1)
	6 (1)	6 (1)	6 (3)	6 (1)
	Lower iliac quadrant	Lower sacrum quadrant	Lower sacrum quadrant	Lower iliac quadrant
Group 4 (10; 29.2]	Right joint		Left joint	
	Upper iliac quadrant	Upper sacrum quadrant	Upper sacrum quadrant	Upper iliac quadrant
	6 (0)	6 (1)	6 (2)	6 (0)
	6 (0)	6 (2)	5 (3)	6 (1)
	Lower iliac quadrant	Lower sacrum quadrant	Lower sacrum quadrant	Lower iliac quadrant

**Table 6:** The results of sensitivity, specificity and accuracy calculated based on the total sum of voxels recognized as TP, TN, FP and FN together with the results of sensitivity calculated from the total amount of lesions recognized as TP and FN by the algorithm.

Groups Based on Deviation Angle		Total Number of Pixels	Total Number of Lesions
Total	Sensitivity	0.76	0.75
	Specificity	0.97	-
	Accuracy	0.97	-
Group 1 [0; 2.2]	Sensitivity	0.58	0.80
	Specificity	0.97	-
	Accuracy	0.96	-
Group 2 (2.2; 5.7]	Sensitivity	0.83	0.78
	Specificity	0.97	-
	Accuracy	0.96	-
Group 3 (5.7; 10]	Sensitivity	0.83	0.77
	Specificity	0.97	-
	Accuracy	0.97	-
Group 4 (10; 29.2]	Sensitivity	0.70	0.66
	Specificity	0.97	-
	Accuracy	0.97	-

**Table 7:** Summary of the most important results ^1^.

		Group 1	Group 2	Group 3	Group 4	Differences between Groups
Bone segmentation	Visual scale [Median (IQR)]	47 (4)	47 (4)	46 (5)	47 (5)	No
	Dice similarity coefficient	0.9839 (0.0016)	0.9825 (0.0012)	0.9795 (0.0019)	0.9819 (0.0014)	Yes ^2^
BME segmentation	Visual scale [Median (IQR)]	42.5 (9)	43 (8)	43 (8)	45 (7)	No
	SPARCC [Median (IQR)]	0 (6.5)	0 (6)	0 (9)	0 (4)	No
	AUC [Value]	0.823	0.917	0.923	0.917	-

^1^ Detailed description is provided in text. ^2^ Post-hoc analysis showed difference between group 1 and group 3.

## Data Availability

Not applicable.
